# Localized SCF and IGF-1 secretion enhances erythropoiesis in the spleen of murine embryos

**DOI:** 10.1242/bio.201410686

**Published:** 2015-04-17

**Authors:** Keai Sinn Tan, Tomoko Inoue, Kasem Kulkeaw, Yuka Tanaka, Mei I Lai, Daisuke Sugiyama

**Affiliations:** 1 Department of Research and Development of Next Generation Medicine, Faculty of Medical Sciences, Kyushu University, Fukuoka 812-8582 Japan; 2 Department of Pathology, Faculty of Medicine and Health Sciences, Universiti Putra Malaysia, 43400 Serdang, Selangor Darul Ehsan, Malaysia; 3 Center for Clinical and Translational Research, Kyushu University Hospital, Fukuoka 812-8582 Japan; 4 Department of Clinical Study, Center for Advanced Medical Innovation, Kyushu University, Fukuoka 812-8582 Japan

**Keywords:** Fetal spleen, Erythropoiesis, Niche, Cytokines

## Abstract

Fetal spleen is a major hematopoietic site prior to initiation of bone marrow hematopoiesis. Morphologic analysis suggested erythropoietic activity in fetal spleen, but it remained unclear how erythropoiesis was regulated. To address this question, we performed flow cytometric analysis and observed that the number of spleen erythroid cells increased 18.6-fold from 16.5 to 19.5 days post-coitum (dpc). Among erythropoietic cytokines, SCF and IGF-1 were primarily expressed in hematopoietic, endothelial and mesenchymal-like fetal spleen cells. Cultures treated with SCF and/or IGF-1R inhibitors showed significantly decreased CD45−c-Kit−CD71+/−Ter119+ erythroid cells and downregulated *Gata1*, *Klf1* and *β-major globin* expression. Administration of these inhibitors to pregnant mice significantly decreased the number of CD45−c-Kit−CD71+/−Ter119+ cells and downregulated *β-major globin* gene expression in embryos derived from these mice. We conclude that fetal spleen is a major erythropoietic site where endothelial and mesenchymal-like cells primarily accelerate erythropoietic activity through SCF and IGF-1 secretion.

## Introduction

Erythropoiesis is defined as the process of generating mature red blood cells (McGrath and Palis, 2008). During mouse embryogenesis, primitive erythropoiesis occurs in the yolk sac from 7.5–8.5 days post-coitum (dpc), and erythroid progenitor cells mature in the circulation and enucleate between 14.5–16.5 dpc (Kingsley et al., 2004; Palis et al., 1999). Definitive erythropoiesis begins in the yolk sac at 9.0 dpc and then shifts to fetal liver, fetal spleen and bone marrow (Bertrand et al., 2005; Houssaint, 1981). Fetal liver serves as the primary organ for erythroid cell expansion and maturation at mid-gestation before bone marrow hematopoiesis initiates (Ayres-Silva et al., 2011; Ema and Nakauchi, 2000; Morrison et al., 1995). Between 14.5–15.5 dpc, fetal liver becomes a less favorable environment for erythropoiesis, as the liver begins to switch from a predominantly hematopoietic to a metabolic function (Guo et al., 2009). Fetal spleen explants cultured *in vitro* produce hematopoietic progenitor and stem cells between 12.5–14.5 dpc, suggesting that hematopoietic cells colonize fetal spleen, which likely fills the hematopoietic “gap” between fetal liver and bone marrow (Godin et al., 1999; Sasaki and Matsumura, 1987).

For over 40 years it has been known that in adults the splenic microenvironment supports erythroid development to a greater extent than myeloid development (Wolf and Trentin, 1968). However, how embryonic spleen hematopoiesis is regulated remains unclear. The spleen is reportedly a site of active myelopoiesis during late embryonic and perinatal stages, and gradually becomes a site of lymphopoiesis after postnatal week one (Ohno et al., 1993). Between 13.5–15.5 dpc, spleen hematopoietic cells are composed primarily of myeloid and erythroid cells (Desanti et al., 2008); however, only a few investigators have analyzed fetal spleen erythropoiesis (Godin et al., 1999). One study showed that at 14.5 dpc fetal spleen stromal cells drive macrophage and B cell commitment (Bertrand et al., 2006). Microscopic observation suggests that the spleen becomes erythropoietic at between 16.0–17.0 dpc until around the first week of postnatal life (Djaldetti et al., 1972; Sasaki and Matsumura, 1988).

Cell fate is determined by intrinsic and extrinsic factors. Our group has characterized embryonic regulation of the mouse hematopoietic niche, a key extrinsic component of the hematopoietic environment (Sugiyama et al., 2011a). Particularly, extrinsic regulation through cytokine secretion, cell-cell interactions and extracellular matrix activity is required for survival, self-renewal, proliferation and differentiation of erythroid cells into mature red blood cells (Watt and Hogan, 2000). Several cytokines, such as erythropoietin (Epo), stem cell factor (SCF), insulin-like growth factor 1 (IGF-1), interleukin 3 (IL-3) and granulocyte-macrophage colony-stimulating factor (GM-CSF), are required for optimal development and terminal differentiation of erythroid cells (Emerson et al., 1989; Goodman et al., 1985; Muta et al., 1994; Umemura et al., 1989). Binding of Epo to its receptor, EpoR, which is expressed on the surface of erythroid progenitors, is particularly critical for these activities (Koury and Bondurant, 1992; Palis, 2014). SCF, a c-Kit ligand, is required for growth of burst-forming unit-erythroids (BFU-Es) under serum-free conditions (Dai et al., 1991). Also, formation of erythrocyte colony-forming units (CFU-Es) requires synergistic SCF and Epo activity (Wu et al., 1997), whereas, IGF-1 stimulates proliferation of erythroid progenitor cells in peripheral blood and bone marrow (Miyagawa et al., 2000).

In this study, we first characterized hematopoietic cell types and identified that erythropoiesis is the dominant activity in fetal spleen at both 16.5 dpc and 19.5 dpc. To investigate extrinsic factors regulating fetal spleen erythropoiesis, we focused on the effect of cytokine secretion by 16.5 dpc fetal spleen cells including hematopoietic, endothelial and unclassified (or mesenchymal-like) cells on erythropoiesis. We found that SCF and IGF-1 are the primary erythropoietic cytokines expressed in fetal spleen. Finally, *in vitro* and *in vivo* analyses using inhibitors of SCF and IGF-1R revealed that both are crucial factors that accelerate spleen erythropoiesis at 16.5 dpc.

## Results

### Characterization of fetal spleen and liver cells

**Fig. 1.**
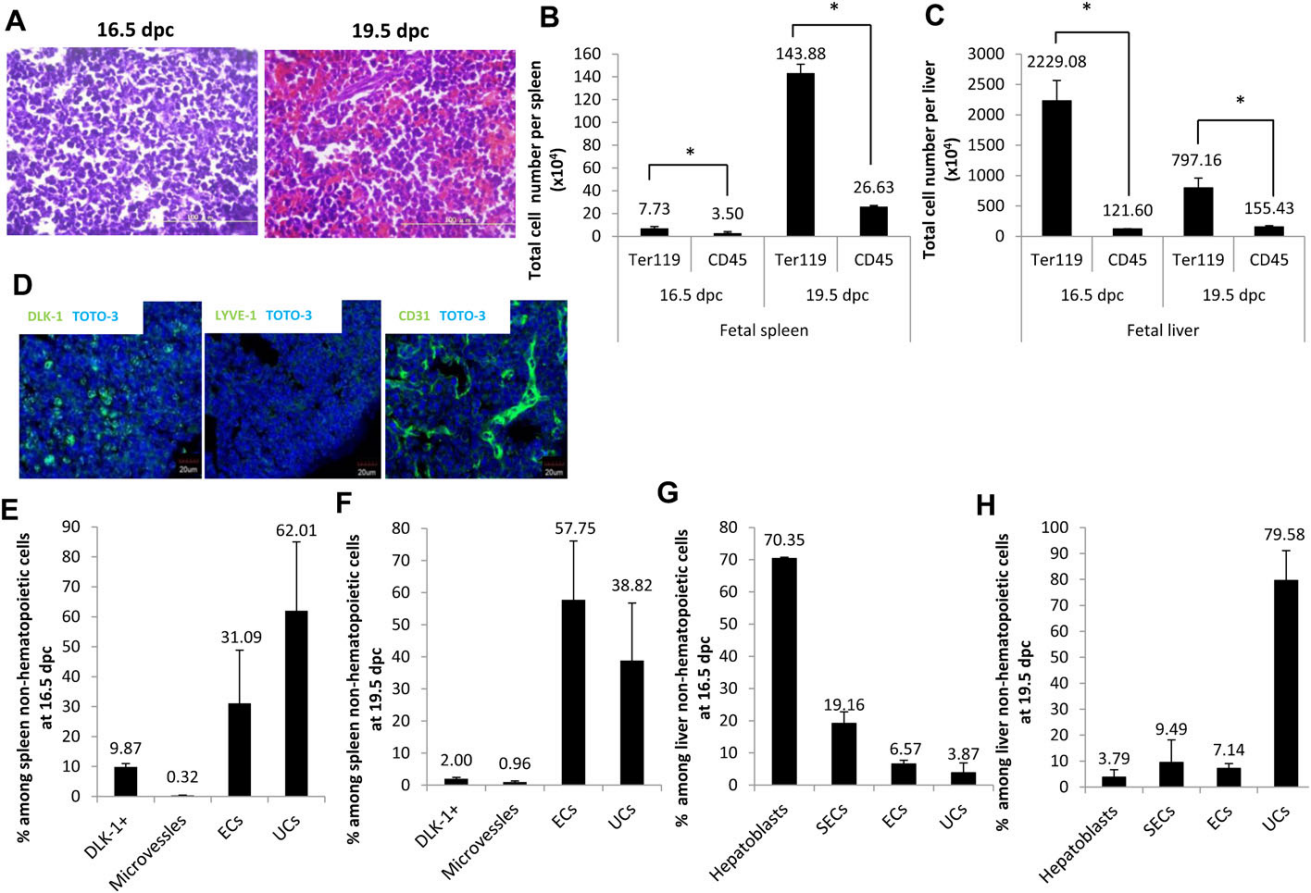
Characterization of fetal spleen and liver cells. (A) Hematoxylin and eosin-stained sections of fetal spleen at 16.5 and 19.5 dpc. Scale bars: 100 µm. (B) Graph showing total number of Ter119+ and CD45+ cells per spleen at 16.5 and 19.5 dpc (n = 3). (C) Graph showing total number of Ter119+ and CD45+ cells per liver at 16.5 and 19.5 dpc (n = 3). (D) Immunohistochemistry staining of CD45−Ter119− non-hematopoietic fetal spleen cells. Spleen sections were prepared from ICR mouse embryos at 16.5 dpc and stained with DLK-1 (green), LYVE-1 (green), CD31 (green) and TOTO-3 (blue). Scale bars: 20 µm. (E,F) Graphs showing percentage of fetal spleen cells among non-hematopoietic cells at 16.5 and 19.5 dpc (n = 3). Data are means±standard deviation (SD). (G,H) Graphs showing percentage of fetal liver cells among non-hematopoietic cells at 16.5 and 19.5 dpc (n = 3). Data are means±standard deviation (SD). See also supplementary material Fig. S1A,B. *P<0.05.

To investigate which lineage commitment is predominant in fetal spleen, we performed hematoxylin and eosin staining at 16.5 dpc and 19.5 dpc. In agreement with previous reports (Djaldetti et al., 1972), at 16.5 dpc we found that the spleen contains blastic cells morphologically defined as small cells with round, dense and deeply basophilic nuclei (Fig. 1A). By 19.5 dpc spleen contained increased numbers of red blood cells morphologically defined as eosinophilic cells lacking nuclei. Next, to quantify erythropoietic activity in spleen after 16.5 dpc, we performed flow cytometry by using the erythroid cell marker Ter119 and the common leukocyte cell marker CD45 (supplementary material Fig. S1A,B). Ter119 marks erythroid cells at various differentiation stages (from early proerythroblasts to mature red blood cells) but does not mark cells exhibiting typical BFU-E and CFU-E activities (Kina et al., 2000). The total number of erythroid cells expressing Ter119 per fetal spleen increased 18.6-fold from 16.5 dpc to 19.5 dpc (Fig. 1B). The number of CD45-positive cells per spleen also increased 7.6-fold from 16.5 to 19.5 dpc. As in fetal liver, the total number of erythroid cells expressing Ter119 per fetal liver decreased 2.7-fold from 16.5 to 19.5 dpc (Fig. 1C), while the number of CD45-positive cells per spleen increased 1.3-fold. Thus, overall, the number of erythroid cells and leukocytes was greater in fetal liver than in fetal spleen. Nonetheless, considering the size of spleen and liver, it is clear that robust erythropoiesis occurs in spleen at these stages. To investigate how spleen erythropoiesis is regulated, we assessed fetal spleen cell cytokine secretion. Delta-like 1 homolog (DLK-1)-expressing hepatoblasts are the primary cells that express SCF and Epo proteins in fetal liver at 14.5 dpc, a time coincident with erythroid cell expansion (Sugiyama et al., 2011b). Hence, for our analysis we used DLK-1 to identify cytokine-secreting cells. We also used staining for platelet endothelial cell adhesion molecule 1 (PECAM-1 or CD31) and lymphatic vessel endothelial hyaluronic acid receptor 1 (LYVE-1) to mark endothelial cells of blood vessels and capillaries, respectively (Neubauer et al., 2000; Tan et al., 2013). Immunohistochemistry revealed that fetal spleen cells express detectable amounts of DLK-1, CD31 and LYVE-1 proteins, suggesting that fetal spleen contains DLK-1-expressing cells and endothelial cells (Fig. 1D). To further characterize fetal spleen cells, we performed flow cytometry. As shown in supplementary material Fig. S1A, fractions of DLK-1-expressing cells, microvessels, endothelial cells and unclassified cells were sorted from fetal spleen at 16.5 dpc and 19.5 dpc based on expression of the following surface markers: (1) CD45−Ter119−DLK-1+ to define DLK-1 expressing cells, (2) CD45−Ter119−LYVE-1+CD31+ to define microvessels, (3) CD45−Ter119−LYVE-1−CD31+ to define endothelial cells, and (4) CD45−Ter119−LYVE-1−CD31− to define unclassified cells. Among CD45−Ter119− non-hematopoietic cells in fetal spleen at 16.5 dpc, 9.9±1.1% were DLK-1-expressing cells, 0.3±0.1% were microvessels, 31.1±17.8% were endothelial cells and 62.0±23.0% were unclassified cells (Fig. 1E). In 19.5 dpc fetal spleen, 2.0±0.4% were DLK-1-expressing cells, 1.0±0.4% were microvessels, 57.8±18.3% were endothelial cells and 38.9±17.9% were unclassified cells (Fig. 1F). As shown in supplementary material Fig. S1B, fractions containing hepatoblasts, sinusoidal endothelial cells, endothelial cells and unclassified cells were sorted from fetal liver at 16.5 and 19.5 dpc based on expression of the following surface markers: (1) CD45−Ter119−DLK-1+ (hepatoblasts), (2) CD45−Ter119−LYVE-1+CD31+ (sinusoidal endothelial cells), (3) CD45−Ter119−LYVE-1−CD31+ (endothelial cells), and (4) CD45−Ter119−LYVE-1−CD31− (unclassified cells). Among CD45−Ter119− non-hematopoietic cells in liver at 16.5 dpc, 70.3±0.4% were hepatoblasts, 19.2±3.6% were sinusoidal endothelial cells, 6.6±1.2% were endothelial cells and 3.9±3.1% were unclassified cells (Fig. 1G). In 19.5 dpc liver, 3.8±2.9% were hepatoblasts, 9.5±8.6% were sinusoidal endothelial cells, 7.1±1.9% were endothelial cells and 79.6±11.5% were unclassified cells (Fig. 1H). Taken together, in 16.5 or 19.5 dpc spleen, the proportion of DLK-1-expressing cells and microvessels was relatively low compared to proportions of endothelial and unclassified cells. In fetal liver, however, hepatoblasts constituted the major population at 16.5 dpc relative to other cell populations, but the percentage of hepatoblasts among non-hematopoietic cells in liver decreased by 19.5 dpc. These results suggest that endothelial and unclassified cells play important roles in erythropoiesis in 16.5 dpc spleen, while hepatoblasts have a more prominent function in erythropoiesis in fetal liver.

### Cytokine expression in fetal spleen cells

**Fig. 2.**
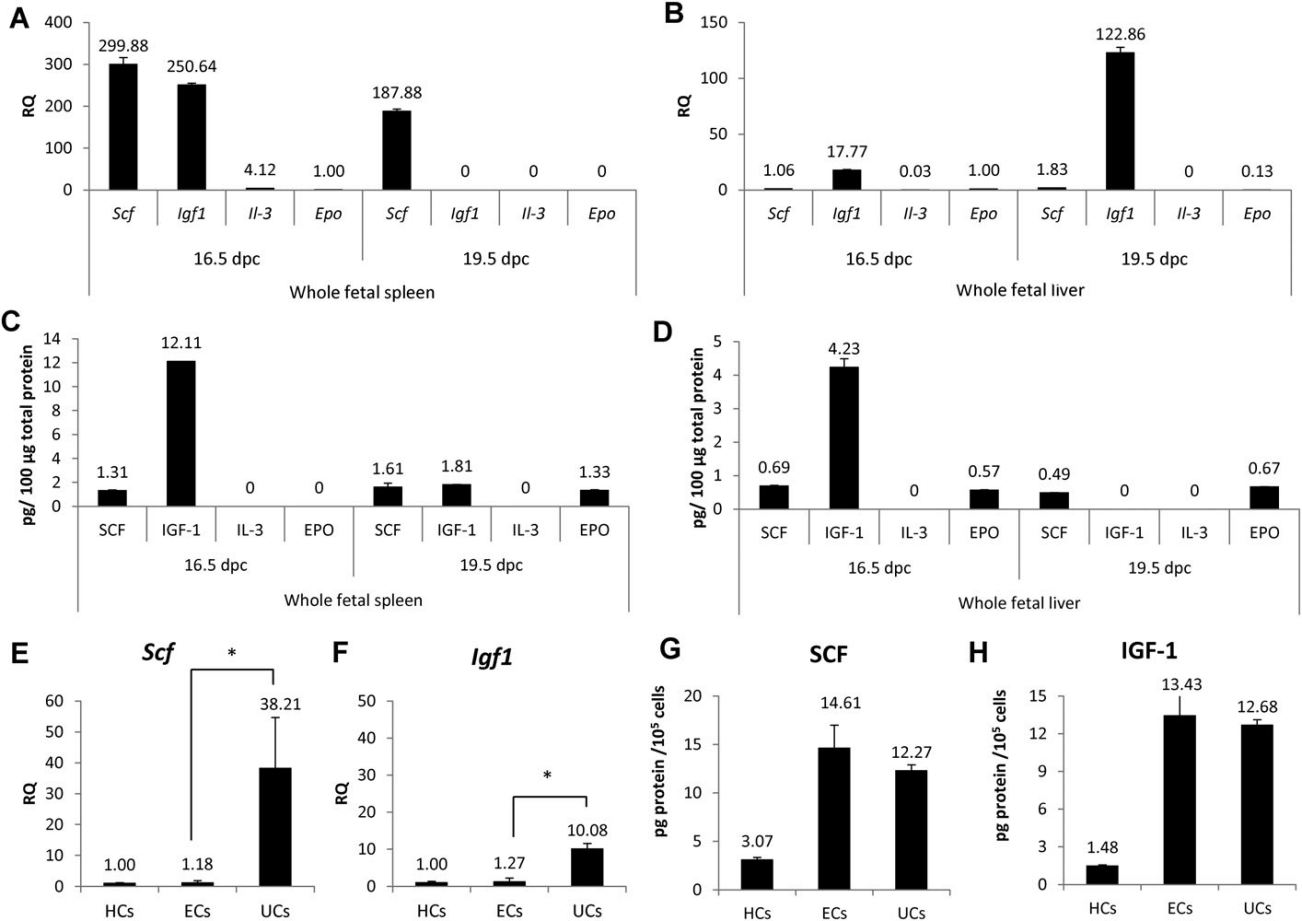
Expression of cytokine mRNA and protein in fetal spleen and liver. (A) Relative expression (RQ) of *stem cell factor* (*Scf*), *insulin-like growth factor1* (*Igf1*), *interleukin-3* (*Il-3*) and *erythropoietin* (*Epo*) mRNAs were examined in whole fetal spleen cells at 16.5 and 19.5 dpc by real-time PCR. *Epo* expression in 16.5 dpc whole fetal spleen served as a control. (B) Relative expression (RQ) of *stem cell factor* (*Scf*), *insulin-like growth* factor1 (*Igf1*), *interleukin-3* (*Il-3*) and *erythropoietin* (*Epo*) mRNAs was determined in whole fetal liver at 16.5 and 19.5 dpc by real-time PCR. *Epo* expression in 16.5 dpc whole fetal spleen served as a control. (C,D) Amounts of SCF, IGF-1, IL-3 and EPO protein per 100 µg total protein in spleen at 16.5 and 19.5 dpc. (D) Amounts of SCF, IGF-1, IL-3 and EPO protein per 100 µg total protein in liver at 16.5 and 19.5 dpc. (E,F) Relative expression (RQ) of *Scf* and *Igf1* mRNAs was determined by real-time PCR in hematopoietic cells (HCs), endothelial cells (ECs) and unclassified cells (UCs) sorted by flow cytometry, according to gates defined in supplementary material Fig. S1A. HCs served as controls. *Scf* and *Igf1* mRNA expression was higher in UCs than in ECs at 16.5 dpc fetal spleen (n = 3). (G,H) Amounts of SCF and IGF-1 protein per 100,000 cells in HCs, ECs, and UCs (n = 3). See also supplementary material Fig. S1E,F. Data are means±standard deviation (SD). *P<0.05.

Given that the total number of erythroid cells per spleen markedly increased from 16.5 dpc to 19.5 dpc, we hypothesized that spleen cells at 16.5 dpc likely accelerate erythropoiesis by providing extrinsic signals. Thus to investigate whether fetal spleen cells express erythropoietic cytokines, we performed real-time PCR to assess *Scf*, *Igf1*, *Il-3* and *Epo* mRNAs in whole spleen cells at 16.5 and 19.5 dpc (Fig. 2A). Both *Scf* and *Igf1* mRNAs were highly expressed at 16.5 dpc, while *Epo* and *Il-3* mRNAs were not. On the other hand, in 19.5 dpc whole spleen cells, *Scf* was highly expressed, while *Igf1*, *Epo* and *Il-3* transcripts were not detected. To compare the microenvironment of whole fetal spleen cells with that of whole fetal liver, we assessed expression of erythropoietic cytokines in 16.5 dpc or 19.5 dpc liver. At 16.5 dpc, *Igf1* was highly expressed in liver, while *Scf*, *Il-3* and *Epo* mRNAs were not (Fig. 2B). We then investigated SCF, IGF-1, IL-3 and EPO protein expression in both spleen and liver at 16.5 and 19.5 dpc by ELISA (Fig. 2C,D). We detected IGF-1 (12.1 pg protein/100 µg total protein) and SCF (1.3 pg protein/100 µg total protein) (Fig. 2C) in whole fetal spleen at 16.5 dpc, while at 19.5 dpc in spleen, we detected SCF (1.6 pg protein/100 µg total protein), IGF-1 (1.8 pg protein/100 µg total protein) and EPO (1.3 pg protein/100 µg total protein). IL-3 protein was not detected in spleen at either 16.5 or 19.5 dpc (Fig. 2C). In 16.5 dpc liver, IGF-1 (4.2 pg protein/100 µg total protein) was highly expressed, while we observed lower levels of SCF (0.7 pg protein/100 µg total protein) and EPO (0.6 pg protein/100 µg total protein) (Fig. 2D). In 19.5 dpc liver, we detected SCF (0.5 pg protein/100 µg total protein) and EPO (0.7 pg protein/100 µg total protein). IL-3 protein was not detected in whole fetal liver at 16.5 or 19.5 dpc, while IGF-1 protein was not detected in 19.5 dpc liver (Fig. 2D) (supplementary material Fig. S2C,D).

To investigate which spleen cell components produce SCF and IGF-1, we used real-time PCR to assess *Scf* and *Igf1* mRNAs in hematopoietic, endothelial and unclassified cells sorted from fetal spleen at 16.5 dpc. As shown in Fig. 2E,F, unclassified cells expressed 32-fold and 8-fold higher levels of *Scf* and *Igf1*, respectively, than did endothelial cells. We then investigated SCF and IGF-1 protein expression by ELISA (Fig. 2G,H). SCF protein was highly expressed in both endothelial cells (14.6 pg protein/100,000 cells) and unclassified cells (12.3 pg protein/100,000 cells) but was expressed to a lesser extent in hematopoietic cells (3.1 pg protein/100,000 cells). Likewise, IGF-1 protein was also expressed in both endothelial cells (13.4 pg protein/100,000 cells) and unclassified cells (12.9 pg protein/100,000 cells) but was expressed to a lesser extent in hematopoietic cells (1.9 pg protein/100,000 cells). Comparison of the amount of SCF and IGF-1 proteins per 100 µg of total protein indicated that protein levels corresponded with mRNA expression (supplementary material Fig. S1E,F). We conclude that spleen erythropoiesis is likely regulated extrinsically by endothelial cells and unclassified cells through SCF and IGF-1 secretion.

### Unclassified cells possess mesenchymal properties

**Fig. 3.**
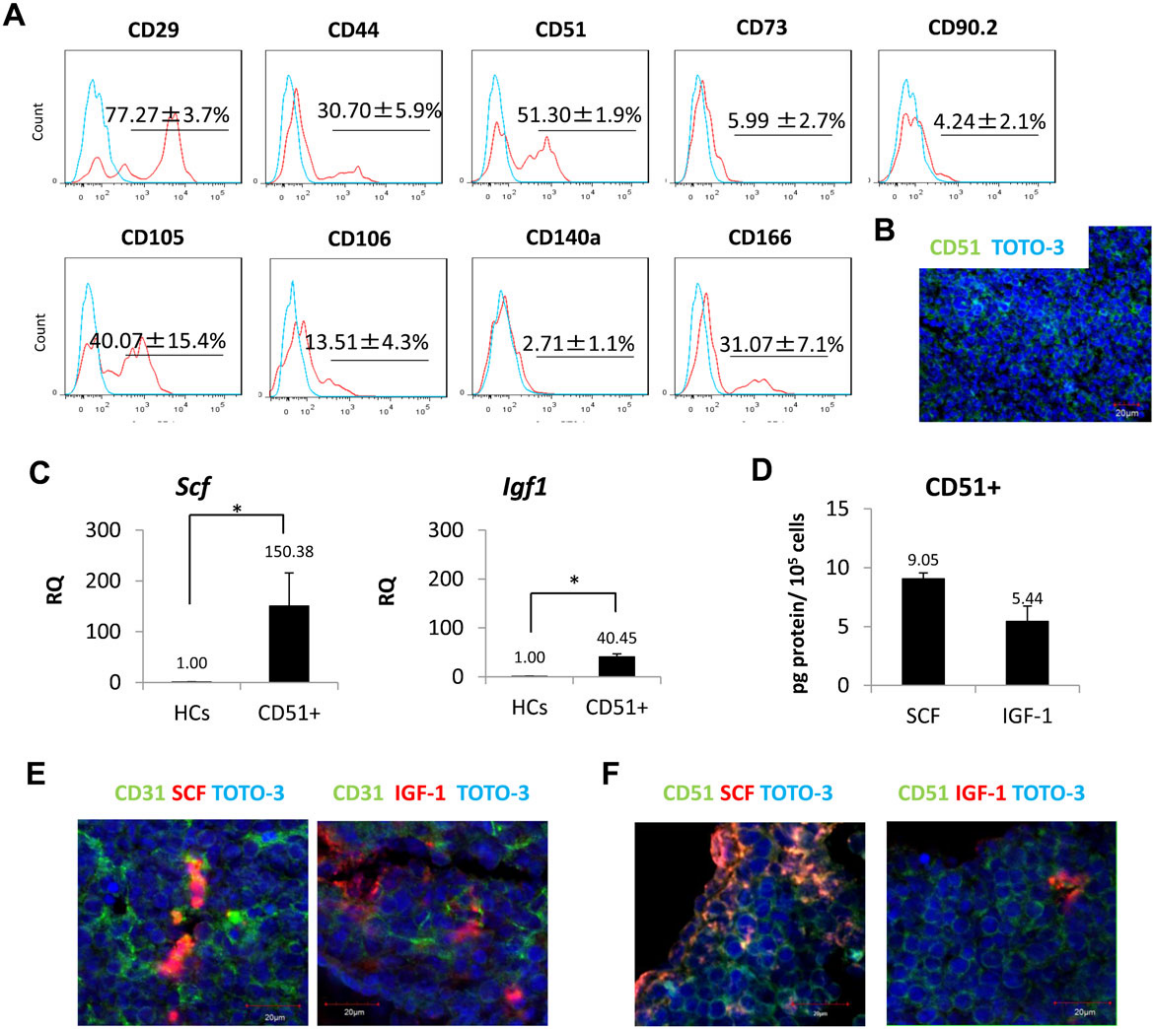
Characterization of unclassified cells (UCs). (A) Analysis of the mesenchymal markers CD29, CD44, CD51, CD73, CD90.2, CD105, CD106, CD140a and CD166 among CD45−Ter119−CD31−LYVE-1− unclassified cells (n = 3). (B) Spleen sections from 16.5 dpc were stained with CD51 (green) and TOTO-3 (blue). Scale bar: 20 µm. (C) A single cell suspension was obtained from fetal spleen at 16.5 dpc. CD45−Ter119−CD31−LYVE-1−CD51+ defines CD51+ cells. Relative expression (RQ) of *Scf* and *Igf1* mRNAs was examined by real-time PCR in CD51+ cells (n = 3). Hematopoietic cells (HCs) served as reference controls. (D) Amounts of SCF and IGF-1 protein per 100,000 cells in CD51+ cells. Expression of SCF and IGF-1 was higher in CD51+ cells than in HCs (n = 3). (E,F) Immunohistochemistry of endothelial and mesenchymal cell markers, CD31 and CD51, respectively stained with SCF and IGF-1 at 16.5 dpc. Samples were observed under confocal microscopy. CD31+/− and CD51+/− cells expressed both SCF and IGF-1. Scale bars: 20 µm. Data are mean±standard deviation (SD). *P<0.05.

To further characterize unclassified cells in fetal spleen, we investigated this population by flow cytometry using additional mesenchymal markers (Giuliani et al., 2011). At present, there is no single surface marker available that serves as an unequivocal marker of mesenchymal cells. Thus, for our study we used several mesenchymal cell markers, including CD29 (integrin β1), CD44 (HCAM), CD51 (integrin αv), CD73 (SH3), CD90.2 (Thy1.2), CD105 (endoglin), CD106 (VCAM-1), CD140a (PDGFRα) and CD166 (ALCAM). Among unclassified cells, the percentage of cells positive for CD29 was 77.3±3.7%; for CD44 was 30.7±5.9%; for CD51 was 51.3±1.9%; for CD73 was 6.0±2.7%; for CD90.2 was 4.2±2.1%; for CD105 was 40.1±15.4%; for CD106 was 13.5±4.3%; for CD140a was 2.7±1.1%; and for CD166 was 31.1±7.1% (Fig. 3A). Overall, these findings suggest that unclassified cells possess mesenchymal properties. Among markers, we paid special attention to CD51, since it is reportedly expressed on bone marrow mesenchymal stem cells (Pinho et al., 2013). Immunohistochemistry revealed that a very large population of CD51 cells in fetal spleen at 16.5 dpc (Fig. 3B). We then performed real-time PCR with sorted CD51+ cells. As shown in Fig. 3C, CD51+ cells expressed higher levels of *Scf* and *Igf1* mRNAs than did hematopoietic cells, which served as controls (supplementary material Fig. S2A,B). ELISA analysis confirmed that CD51+ cells express SCF and IGF-1 protein (Fig. 3D; supplementary material Fig. S2C). Immunohistochemistry also showed that CD31+/− and CD51+/− cells express SCF and IGF-1 proteins (Fig. 3E,F), suggesting that both endothelial cells and unclassified cells with mesenchymal properties likely secrete SCF and IGF-1 secretion in fetal spleen.

### Spleen stromal cells accelerate erythropoiesis *in vitro*

**Fig. 4.**
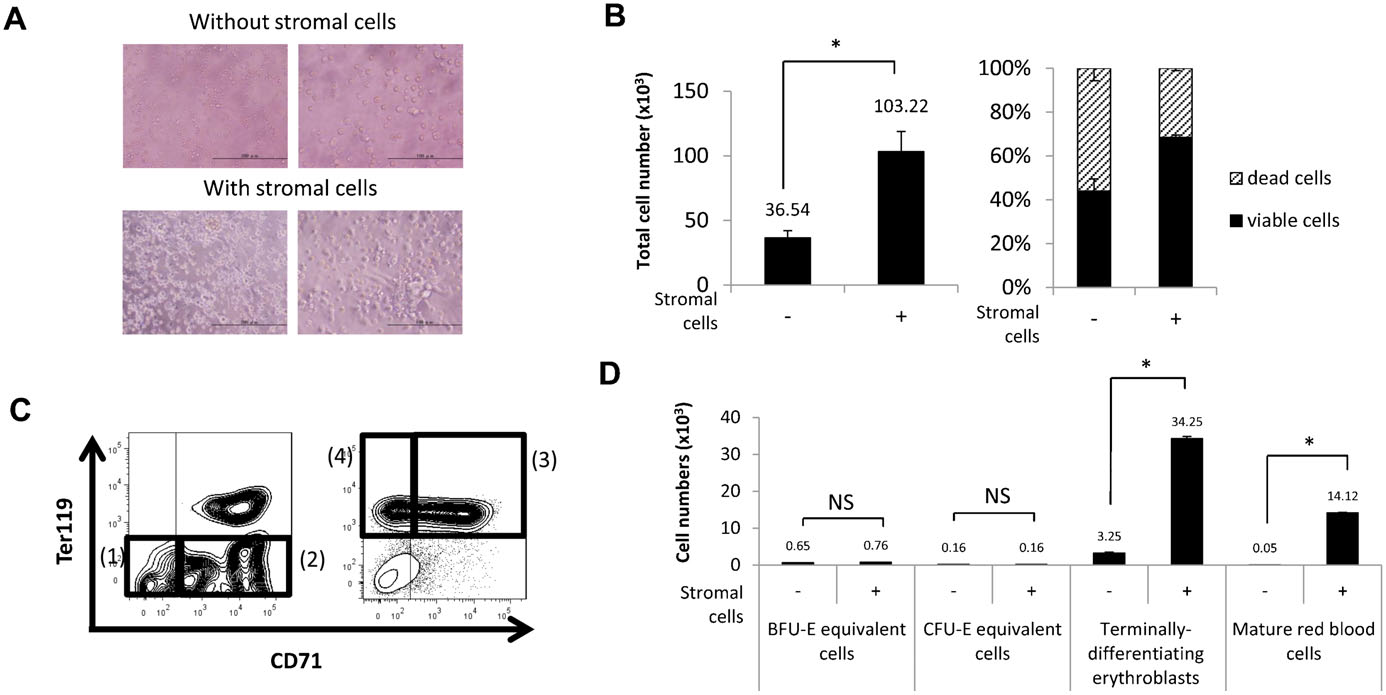
Acceleration of erythropoiesis in hematopoietic cells cultured with stromal cells. (A) Microscopic images after 24 hours of culture of hematopoietic cells with or without stromal cells. Cells that adhered to a plate after two hours were used as stromal cells. Hematopoietic cells were cultured either alone or in co-culture with stromal cells. Scale bars: left, 200 µm; right, 100 µm. (B) Graphs showing the total number of cells (left) and the percentage of viable and dead cells (right) in conditions with or without stromal cells. Both the total number and percentage of viable cells were higher among cells cultured with stromal cells (n = 3). (C) Gating strategy of flow cytometric analysis. (1) CD45−c-Kit+CD71−Ter119− defines BFU-E equivalent cells; (2) CD45−c-Kit+CD71+Ter119− defines CFU-E equivalent cells; (3) CD45−c-Kit−CD71+Ter119+ defines terminally-differentiating erythroid cells; and (4) CD45−c-Kit−CD71−Ter119+ defines mature red blood cells. See also supplementary material Fig. S3A,B. (D) Graphs showing the number of BFU-E equivalent cells, CFU-E equivalent cells, terminally-differentiating erythroid cells and mature red blood cells after 24 hours of culture with or without stromal cells. The number of terminally-differentiating erythroid cells and mature red blood cells increased in cultures including stromal cells (n = 3). Data are means±standard deviation (SD). NS, not significant. *P<0.05.

To investigate the function of stromal cells comprising the niche in fetal spleen erythropoiesis we undertook co-culture analysis. To do so we dissociated whole fetal spleen cells into single cells and cultured them on 0.1% gelatin-coated plates. After two hours, cells that adhered to the plates were used as stromal cells, while non-adherent cells, which contained hematopoietic cells, were collected and cultured either alone or in co-culture with the stromal cells. After 24 hours of either co-culture or culture of hematopoietic cells alone, we observed cells resembling fibroblasts adhering to the plate (Fig. 4A). There were hematopoietic cells found among non-adherent cells and that total number of non-adherent cells cultured with stromal cells had increased in number 2.8-fold relative to cells cultured without stromal cells (Fig. 4B, left). Also, when we assessed cell viability by calculating the percentage of propidium iodide-negative cells by flow cytometry, we found that the viability of cells cultured for 24 hours with stromal cells was 68.5%, while that of cells cultured without stromal cells was 43.9% (Fig. 4B, right).

Next, we used flow cytometry to assess erythroid cells that had been co-cultured with stromal cells versus those grown without stromal cells. We measured the erythroid cell population from fetal spleen by flow cytometry using the following criteria: (1) CD45−c-Kit+CD71−Ter119− defined BFU-E equivalent cells; (2) CD45−c-Kit+CD71+Ter119− defined CFU-E equivalent cells; (3) CD45−c-Kit−CD71+Ter119+ defined terminally-differentiating erythroid cells (supplementary material Fig. S3A); and (4) CD45−c-Kit−CD71−Ter119+ defined mature red blood cells (supplementary material Fig. S3B) (Inoue et al., 2011; Socolovsky et al., 2001) (Fig. 4C). The number of BFU-E and CFU-E equivalent cells was comparable among cells cultured with or without stromal cells. The number of terminally-differentiating erythroid cells increased 10-fold and that of mature red blood cells increased 282-fold in cells cultured with stromal cells compared to those cultured without stromal cells (Fig. 4D). These findings suggest that fetal spleen erythropoiesis is accelerated through an extrinsic signal derived from stromal cells *in vitro*.

### SCF and IGF-1 accelerate fetal spleen erythropoiesis *in vitro*

**Fig. 5.**
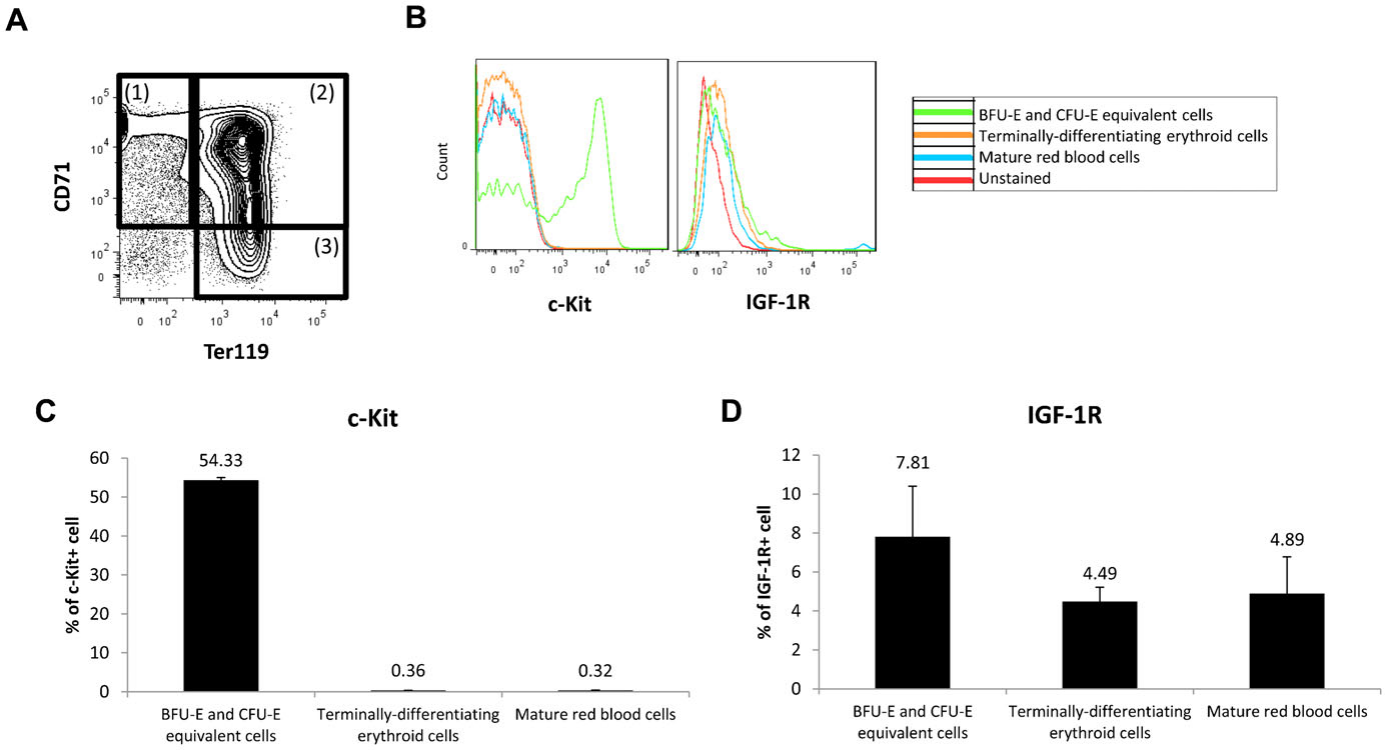
Expression of c-Kit and IGF-1R on erythroid cells. (A) Gating strategy of flow cytometric analysis of c-Kit+ and IGF-1R+ cells during erythropoiesis. (1) CD45−CD71+Ter119− defines BFU-E and CFU-E equivalent cells, (2) CD45−CD71+Ter119+ defines terminally-differentiating erythroid cells, and (3) CD45−CD71−Ter119+ defines mature red blood cells. (B) Representative diagram of flow cytometric analysis showing cell surface expression of c-Kit and IGF-1R on erythroid cells. (C,D) Graphs showing the percentage of c-Kit+ or IGF-1R+ cells in BFU-E and CFU-E equivalent cells, terminally-differentiating erythroid cells and mature red blood cells (n = 3). c-Kit+ and IGF-1R+ cells were expressed primarily in BFU-E and CFU-E equivalent cells. Data are means±standard deviation (SD).

To identify cell types regulated by SCF and IGF-1 signals, we evaluated expression of the SCF receptor (c-Kit) and the IGF-1 receptor (IGF-1R) on erythroid cells in fetal spleen using flow cytometry (Fig. 5A,B). The erythroid cell population was analyzed using the following gating strategy: (1) CD45−CD71+Ter119− defined BFU-E and CFU-E equivalent cells; (2) CD45−CD71+Ter119+ defined terminally differentiating erythroid cells; and (3) CD45−CD71−Ter119+ defined mature red blood cells. Of BFU-E and CFU-E equivalent cells, 54.3% were c-Kit+ cells and 7.8% were IGF-1R+. Of terminally-differentiating erythroid cells only 0.4% were c-Kit+ cells and 4.5% were IGF-1R+, while of mature red blood cells, 0.3% were c-Kit+ and 4.9% were IGF-1R+ (Fig. 5C,D). These observations indicate most c-Kit+ and IGF-1R+ cells are BFU-E and CFU-E equivalent cells.

**Fig. 6.**
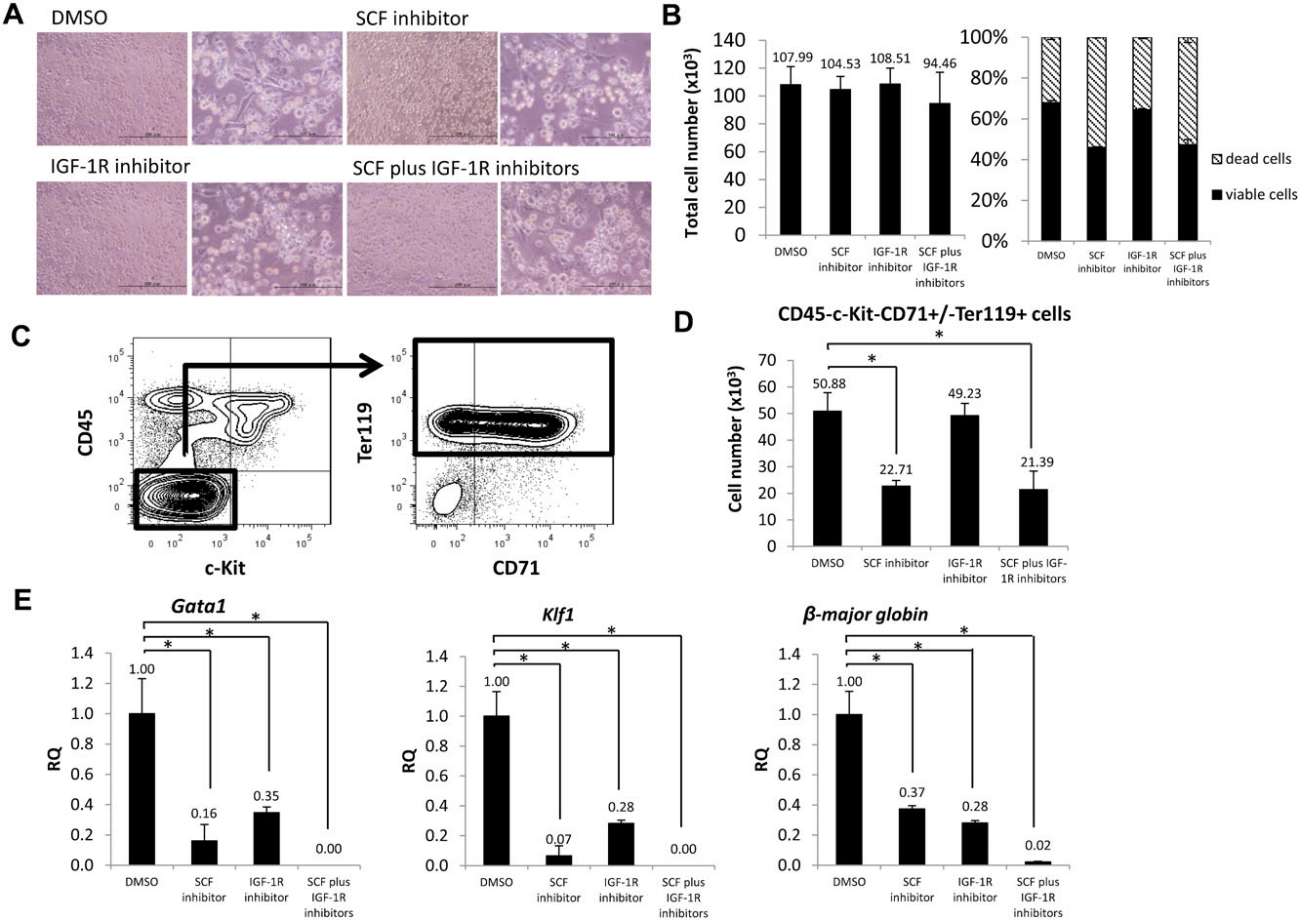
SCF and IGF-1 accelerate fetal spleen erythropoiesis in vitro. (A) Microscopic images of whole fetal spleen cell cultures after 24 hours of treatment with DMSO, an SCF inhibitor, an IGF-1R inhibitor or both. Scale bars: left, 200 µm; right, 100 µm. (B) Graphs showing the total number of cells (left) and percentage of viable and dead cells (right) in culture with those inhibitors (n = 3). (C) Gating strategy of flow cytometric analysis. CD45−c-Kit−CD71+/−Ter119+ defines terminally-differentiating erythroid cells and mature red blood cells. (D) Graph showing the number of terminally-differentiating erythroid cells and mature red blood cells in culture with DMSO, the SCF inhibitor, the IGF-1R inhibitor or both (n = 3). (E) Expression of erythroid mRNAs such as *Gata1*, *Klf1* and *β-major globin* in sorted CD45− cells by real-time PCR. CD45− cells from DMSO-treated cultures served as controls (n = 3). Data are means±standard deviation (SD). *P<0.05.

To determine if SCF and IGF-1 accelerate fetal spleen erythropoiesis, we cultured whole fetal spleen cells with or without an SCF inhibitor, an IGF-1R inhibitor, a combination of the two or a DMSO vehicle control for 24 hours (Fig. 6A) and evaluated cell number and viability (Fig. 6B). The total cell number did not differ among all conditions, while cell viability decreased in cultures treated with the SCF inhibitor (46.0%) and cells treated with both inhibitors (47.3%) compared to cells cultured with DMSO (67.9%) or with the IGF-1R inhibitor (64.4%).

Next, we used flow cytometry to measure the erythroid cell population from cultured whole fetal spleen cells grown in the presence of an SCF inhibitor, an IGF-1R inhibitor, a combination of the two or DMSO vehicle, using the gating strategy shown in Fig. 6C. CD45−c-Kit+CD71+/−Ter119+ cells were defined as terminally-differentiating erythroid cells and mature red blood cells. The number of CD45−c-Kit−CD71+/−Ter119+ cells decreased 2.2-fold in cultures treated with the SCF inhibitor and 2.4-fold in cultures treated with both compared to DMSO control cells (Fig. 6D). To further evaluate altered gene expression in cells cultured with inhibitors, we assessed *Gata1*, *Klf1* and *β-major globin* mRNA by real-time PCR in CD45− cells (Fig. 6E). *Gata1*, *Klf1* and *β-major globin* expression decreased relative to the DMSO control in cultures treated with the SCF inhibitor or the IGF-1R inhibitor. In cells treated with both, *Gata1* and *Klf1* expression was undetectable, whereas that of *β-major globin* expression decreased to only 2% of that of controls. Collectively, treatment with both inhibitors abrogated expression of erythroid genes, suggesting that SCF and IGF-1 are required for fetal spleen erythropoiesis *in vitro*.

### SCF and IGF-1 accelerate fetal spleen erythropoiesis *in vivo*

**Fig. 7.**
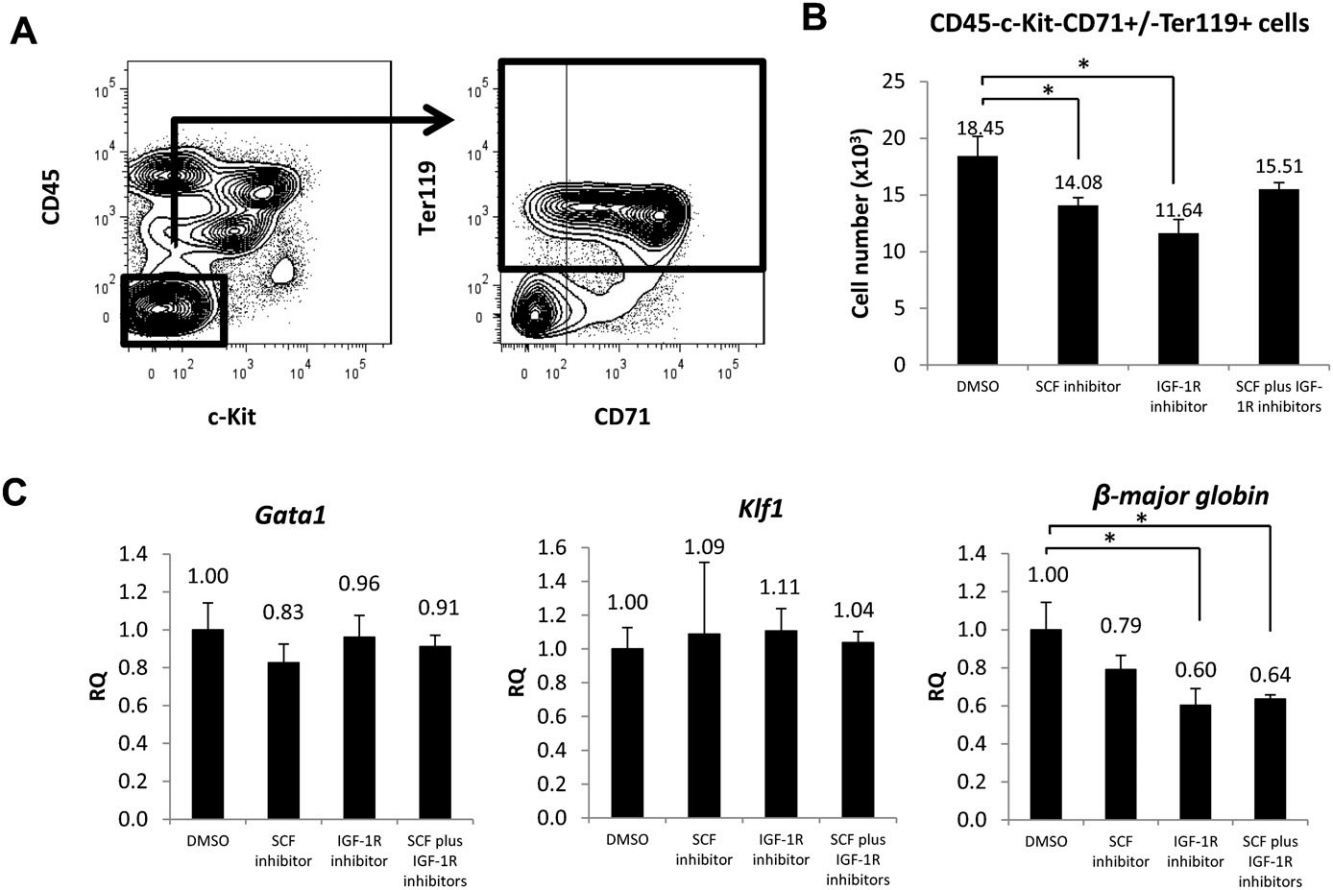
SCF and IGF-1 accelerate fetal spleen erythropoiesis *in vivo*. (A) Gating strategy of flow cytometric analysis. CD45−c-Kit−CD71+/−Ter119+ defines terminally-differentiating erythroid cells and mature red blood cells. (B) Graph showing the number of terminally-differentiating erythroid cells and mature red blood cells in mice treated with DMSO, the SCF inhibitor, the IGF-1R inhibitor or both, two hours after injection (n = 3). (C) Relative expression (RQ) of erythroid genes such as *Gata1*, *Klf1* and *β-major globin* mRNAs in CD45− cells was examined by real-time PCR. CD45− cells from mice injected with DMSO served as controls (n = 3). Data are means±standard deviation (SD). *P<0.05.

To examine SCF and IGF-1 effects *in vivo*, inhibitors (either alone or in combination) or DMSO alone was administered intravenously into pregnant ICR mice at 16.5 dpc. Two hours after inhibitor injection, injected mice were sacrificed and the spleens of their embyros were dissected for flow cytometry analysis of the erythroid cell population. The gating strategy for that analysis is shown in Fig. 7A. Two hours after inhibitor injection, the number of CD45−c-Kit−CD71+/−Ter119+ cells decreased 1.3-fold in SCF inhibitor-treated mouse embryos spleen, 1.6-fold in IGF-1R treated mouse embryos spleen, and 1.2-fold in mouse embryos spleen treated with both inhibitors relative to DMSO controls (Fig. 7B). To further evaluate gene expression *in vivo*, we assessed *Gata1*, *Klf1* and *β-major globin* mRNAs by real-time PCR in CD45− cells sorted from fetal spleen of the embryos of injected mice (Fig. 7C). *Gata1* and *Klf1* expression did not differ significantly in fetal spleen cells taken from mice embryos treated with inhibitors or DMSO vehicle, whereas *β-major globin* expression decreased relative to the DMSO control in tissues taken from mice treated with the SCF inhibitor, the IGF-1R inhibitor or both.

## Discussion

In mice, fetal spleen forms from 11.5–13.5 dpc and becomes hematopoietic at 12.5–13.0 dpc, after initiation of fetal liver hematopoiesis (Godin et al., 1999; Green, 1967; Patterson et al., 2000; Wolber et al., 2002). As an embryo develops, different types of hematopoietic cells reside in spleen. In 15.0 dpc embryos, 40% of spleen cells are F4/80+Mac-1+, indicating that they are macrophage cells (Bertrand et al., 2006). By one week after birth, Mac-1-positive cell reportedly decreases in spleen, whereas cells expressing the lymphoid marker B220 increase in number (Ohno et al., 1993). Furthermore, dendritic cells develop in spleen by postnatal day 4 (Hinton et al., 2011). Taken together, embryonic and perinatal spleen can support development of different cell lineages, particularly myeloid and lymphoid lineages. From 16.5 dpc to 19.5 dpc, we observe that spleen becomes increasingly erythropoietic, and flow cytometric analysis enabled us to quantitate fetal spleen cells, including hematopoietic cells and non-hematopoietic cells during this period. Our analysis suggests that fetal spleen is an erythropoietic organ by 16.5 dpc, a finding compatible with previous reports (Djaldetti et al., 1972; Sasaki and Matsumura, 1988).

Several reports have shown that a hematopoietic environment comprised of stromal cells functions as a niche to support hematopoietic cells. For example, OP-9, a representative stromal cell line established from osteopetrotic mice that lack macrophage colony-stimulating factor, reportedly can sustain hematopoiesis, including erythropoiesis (Kodama et al., 1994; Nakano et al., 1994). The fetal spleen environment supports macrophage and B cell development *in vitro* at 14.5 dpc (Bertrand et al., 2006). Mouse stromal cell lines established from 14.5 dpc spleen secrete macrophage colony-stimulating factor, which accelerates macrophage development (Bertrand et al., 2006). In addition, spleen stromal cell supernatants contain the anti-inflammatory cytokine transforming growth factor-β1, which inhibits T cell proliferation (Li et al., 2006). Stromal cell lines derived from new born mouse spleen support proliferation and differentiation of erythroid cells in the presence of EPO when co-cultured with hematopoietic cells derived from 13.5 dpc fetal liver or adult bone marrow (Yanai et al., 1989). In this study, we characterized stromal cell components by flow cytometry and demonstrated that sorted endothelial and mesenchymal-like cells express the erythropoietic cytokines SCF and IGF-1 at 16.5 dpc. Hematopoietic cells co-cultured with stromal cells of fetal spleen in the presence of an SCF inhibitor or inhibitors of both SCF and IGF-1R exhibited decreased cell viability compared to controls or cells cultured with an IGF-1R inhibitor alone, suggesting that SCF supports erythroid cell survival in fetal spleen *in vitro*.

Fetal liver and spleen are the sites where erythropoiesis occurs (Godin et al., 1999; Sugiyama et al., 2011a). In the fetal liver, mechanisms underlying that hematopoietic stem cell expansion have been well characterized with special attention to niche cells expressing cytokines such as SCF, thrombopoietin, IGF-2 and angiopoietin-like proteins 2 and 3 (Chou and Lodish, 2010; Zhang et al., 2006; Zhang and Lodish, 2004). Previously, our group reported that DLK-1+ hepatoblasts support fetal liver hematopoiesis, particularly erythropoiesis, through EPO and SCF secretion. Loss of DLK-1+ hepatoblasts in *Map2k4*−/− mouse embryos resulted in decreased numbers of hematopoietic cells in fetal liver (Sugiyama et al., 2011b). Here, we showed that SCF and IGF-1 are expressed primarily in fetal spleen endothelial and mesenchymal-like cells and to lesser extent in hematopoietic cells. Among fetal spleen erythroid cells, c-Kit expression decreased with maturation, and IGF-1R expression increased. Our *in vitro* data revealed that addition of an SCF inhibitor to cell cultures decreased the number of CD45−c-Kit−CD71+/−Ter119+ cells, while treatment with an IGF-1R inhibitor did not (Fig. 6D). However, *in vivo* we found that treatment with either inhibitor significantly decreased the number of CD45−c-Kit−CD71+/−Ter119+ cells in fetal spleen (Fig. 7B). This difference might be due to the influence of cytokines secreted from non-splenic tissues. SCF is a key cytokine that promotes differentiation of erythroid progenitors expressing its receptor, c-Kit, in fetal liver and spleen. Embryos deficient in the regulatory subunit of class IA phosphatidylinositol 3-kinase, which functions in c-Kit receptor signaling, reportedly exhibit decreased numbers of BFU-E and CFU-E in fetal liver compared to wild-type controls (Munugalavadla and Kapur, 2005). Here, we demonstrate that blocking SCF signaling via an inhibitor decreases the number of erythroid cells in *vitro* and *in vivo*, suggesting that SCF signaling also regulates erythropoiesis in fetal spleen.

Our previous report showed that SCF is primarily expressed in fetal liver hepatoblasts, suggesting that hepatoblasts are key regulators for hematopoiesis in fetal liver (Sugiyama et al., 2011b). However, the present study shows that SCF is expressed in both endothelial cells and mesenchymal-like cells of fetal spleen, but not in DLK-1-positive cells, suggesting that hematopoietic regulation differs between fetal liver and spleen. At 16.5 dpc *Epo* gene expression is lower in whole fetal spleen than in fetal liver (Sugiyama et al., 2011b). Furthermore, EPO protein expression was detectable in fetal liver but not in fetal spleen at 16.5 dpc, suggesting that EPO is recruited from fetal liver to spleen at this stage (Fig. 2C,D; supplementary material Fig. S1D). As embryos develop, both fetal spleen and liver express EPO (Fig. 2C,D). The source of EPO at later embryonic stages will be investigated in the future.

Previous investigators have shown that at the BFU-E stage of maturation, cells cannot survive without EPO signaling (Udupa and Reissmann, 1979). Here, however, our *in vitro* cultures did not contain exogenous EPO, which suggests that adherent cells in the co-culture provide the EPO survival signal. Cells used in co-culture experiments reported here were derived from whole spleen cells and are likely a heterogeneous population. Macrophages reportedly form erythroblastic islands, regulate erythropoiesis, and produce EPO (Ji et al., 2008; Ohls et al., 1994; Sadahira et al., 2000), suggesting the possibility that macrophages in our co-culture system provided survival signals such as EPO. Thus although co-culture approaches enable interactions with target cells, their utility in identifying critical cell subpopulations is limited due to their heterogeneity.

Mesenchymal stem cells, which are multi-potent precursors present in the stromal fraction of many tissues, can differentiate into osteoblasts, adipocytes and chondrocytes after *in vitro* expansion (Wang et al., 2006). Mesenchymal stem cells that reside in bone marrow could maintain stemness and proliferation of hematopoietic stem cells, as well as support hematopoietic stem cell transplantation (Li and Wu, 2011). Lin−CD140ab+ mesenchymal cells isolated from adult bone marrow tissues express stem/progenitor cell markers such as CD29, CD51, CD73, CD105, CD146, and Sca-1 (Suire et al., 2012). Lin−CD140ab+ cells possess multi-lineage differentiation capacity, as they can form bone *in vivo* and also possess chondrogenic and adipogenic activities, suggesting that they are mesenchymal stem cells. CD51 and CD140a are expressed on Nestin+ mesenchymal stem cells of the bone marrow (Houlihan et al., 2012; Pinho et al., 2013). CD51+/CD140a+ cells express genes functioning in hematopoietic stem cell maintenance and are likely major constituents of the bone marrow hematopoietic stem cell niche (Pinho et al., 2013). By comparison, CD51+ cells among unclassified cells of fetal spleen express *Nestin*, suggesting that they exhibit mesenchymal stem cell characteristics in that milieu (supplementary material Fig. S4A,B). Spleen CD51+ cells express both SCF and IGF-1, suggesting that these factors function in hematopoietic stem cell maintenance and erythropoiesis.

Taken together, our work shows that robust erythropoiesis occurs in fetal spleen that its niche cells accelerate erythropoietic activity through SCF and IGF-1 secretion, and that erythropoietic regulation differs between fetal spleen and liver. Further investigation of erythropoietic regulation should facilitate our understanding of erythropoietic differentiation from pluripotent stem cells and be useful for future regenerative medicine approaches.

## Materials and Methods

### Mice

ICR mice were purchased from Nihon SLC (Hamamatsu). Noon on the day of the plug was considered to be 0.5 day post-coitum (dpc). Animals were handled according to Guidelines for Laboratory Animals of Kyushu University. This study was approved by the Animal Care and Use Committee, Kyushu University (Approval ID: A21-068-0).

### Hematoxylin and eosin staining

Fetal spleens at 16.5 dpc and 19.5 dpc were dissected in PBS under a Leica MZ7.5 stereomicroscope, fixed in 2% paraformaldehyde/PBS at 4°C overnight, washed 3 times with PBS, equilibrated with 30% sucrose/PBS, embedded in OCT compound (SAKURA, Tokyo, Japan) and frozen in liquid nitrogen. Tissues were then sliced at 10 µm thick using a Leica CM1900 UV cryostat, transferred to glass slides (Matsunami, Osaka, Japan) and dried thoroughly. Slides were put into a glass chamber, washed with running tap water for 3 minutes and then stained with Mayer's Hematoxylin solution (Muto Pure Chemicals Co., Ltd., Tokyo, Japan) for 5 minutes to visualize dark nuclei. Slides were then washed under warm running tap water in a glass chamber for 3 minutes and then stained with 1% Eosin Y solution (Wako Pure Chemical Industries, Ltd., Osaka, Japan) for 5 minutes to visualize red blood cells. Slides were again washed in tap water in a glass chamber to remove excess Eosin Y. The slides were successively dehydrated in 95% ethanol, 100% ethanol and then xylene. Tissues were mounted on slides using MGK-S (Matsunami Glass Ind., Ltd., Osaka, Japan), covered with cover glass (Matsunami Glass Ind., Ltd., Osaka, Japan) and stored at room temperature. Fetal spleen morphology was assessed using an Olympus CKX41 inverted microscope, an Olympus DP71 microscope digital camera and image capture software (DP manager version 3.1.1.208 and DP controller 3.2.1.276, Olympus, Tokyo, Japan).

### Cell preparation

Fetal spleens or livers at 16.5 dpc and 19.5 dpc were dissected as described above. To prepare single cell suspensions, fetal spleens or livers were incubated with 3 mg/mL collagenase type 1 (Worthington Biochemical Corporation, New Jersey) in medium supplemented with 10% fetal bovine serum for 30 minutes at 37°C and passed through 70-µm nylon cell strainers (BD Biosciences, California).

### Flow cytometry and cell sorting

Antibodies used for analysis were: Pacific Blue-, APC- and PE-Cy7-conjugated anti-mouse CD45; PE-Cy7-, APC-Cy7- and APC-conjugated anti-mouse Ter119; PE- and APC-conjugated anti-mouse CD31; APC-conjugated anti-mouse LYVE-1; FITC-conjugated anti-mouse DLK-1; PE-Cy7-conjugated anti-mouse CD106; PE-conjugated anti-mouse CD51; PE-conjugated anti-mouse CD73; PE-conjugated anti-mouse CD105; PE-conjugated anti-mouse CD166; PE-conjugated anti-mouse CD44; FITC-conjugated anti-mouse CD29; FITC-conjugated anti-mouse CD90.2; Biotin-conjugated CD144a; FITC-conjugated streptavidin; FITC-conjugated anti-mouse c-Kit; rabbit-anti-mouse IGF-1R; Alexa Fluor®488 donkey anti-rabbit IgG ; PE-conjugated anti-mouse Sca-1; APC-conjugated anti-mouse c-Kit and FITC-conjugated anti-mouse CD71. Antibodies were purchased from eBioscience, San Diego, CA; BioLegend, San Diego, CA; Invitrogen, Carlsbad, CA; and MBL, Nagoya, Japan. Flow cytometric analysis and cell sorting were carried out using a FACSAria SORP cell sorter (BDIS, San Jose, CA). Data files were analyzed using FlowJo software (Tree Star, Inc., San Carlos, CA).

### RNA extraction and real-time PCR analysis

Total RNA was isolated using the RNAqueous®-4PCR kit (Ambion Inc., Austin, Texas) and a RiboPure™ RNA Purification Kit (Ambion Inc.). mRNA was reverse transcribed using a high-capacity RNA-to-cDNA kit (Life Technologies, Carlsbad, CA). Gene expression levels were measured by real-time PCR (StepOnePlus™ real-time PCR; Life Technologies) with TaqMan® Gene Expression Master Mix (Life Technologies). Primers and probes for *Stem cell factor* (*Scf*) (Mm00442972_m1), *Insulin-like growth factor 1* (*Igf1*) (Mm00439560_m1), *Interleukin-3* (*Il-3*) (Mm00439631_m1), *Erythropoietin* (*Epo*) (Mm00433126_m1), *GATA-binding factor 1* (*Gata1*) (Mm01352636_m1), *Kruppel-like factor 1* (*Klf1*) (Mm00516096_m1) and *β-major globin* (*Hbb-b1*) (Mm01611268_g1) were from TaqMan® Gene Expression Assays (Life Technologies). The thermal protocol was set to 2 minutes at 50°C, followed by 10 minutes at 95°C, and then 40 cycles of 15 seconds at 95°C and 1 minute at 60°C. *β-actin* (Mm00607939_s1) served as internal control. All analyses were performed in triplicate; mRNA levels were normalized to *β-actin* and relative expression (RQ) was compared with a reference sample.

### Enzyme-linked immunosorbent assay (ELISA)

Lysates of whole fetal spleen or liver tissues were prepared using the Qproteome Mammalian Protein Prep Kit (Qiagen, Hilden, Germany), according to the manufacturer's instructions. Hematopoietic cells (CD45+/CD45+Ter119+/Ter119+), endothelial cells (CD45−Ter119−CD31+LYVE-1−), unclassified cells (CD45−Ter119−CD31−LYVE-1−) and CD51+ cells among unclassified cells (CD45−Ter119−CD31−LYVE-1−CD51+) were sorted. Lysates were prepared using M-PER Mammalian Protein Extraction Reagent (Thermo Fisher Scientific, Rockford, IL) and a protease inhibitor from the Qproteome Mammalian Protein Prep Kit (Qiagen). Samples were centrifuged at 12,000 rpm for 10 minutes at 4°C. Supernatants containing soluble protein were collected and protein concentration was quantitated using the Bradford reagent (Bio Rad, Hercules, CA) according to the manufacturer's instruction. The optical density (O.D.) at 540 nm was measured using a Thermo Multiskan FC plate reader (Thermo Fisher Scientific). SCF, IGF-1, IL-3 and EPO ELISA assays were conducted using a mouse SCF Quantikine ELISA kit (R&D Systems, Minneapolis, MN), a mouse IGF-1 Quantikine ELISA kit (R&D Systems), a mouse IL-3 Quantikine ELISA kit (R&D Systems) and a mouse EPO Quantikine ELISA kit (R&D Systems), according to the manufacturer's instructions. O.D.s at 450 nm and 540 nm were measured using a Thermo Multiskan FC plate reader.

### Immunohistochemistry

Frozen blocks of 16.5 dpc fetal spleen at were prepared, and 10 µm sections were cut using a Leica CM1900 UV cryostat, transferred to glass slides (Matsunami, Osaka, Japan) and dried thoroughly. Sections were washed three times with PBS, blocked with 1% BSA in PBS and incubated with appropriate dilutions of the following primary antibodies at 4°C overnight: 1:300 anti-mouse CD31 rat IgG (MEC13.3; BD Biosciences, San Diego, CA), 1:300 anti-mouse CD51 rat IgG (RMV-7; BD Biosciences), 1:300 anti-mouse LYVE-1 rat IgG (ALY7; MBL, Woburn, MA) and 1:300 anti-mouse DLK-1 rat IgG (24-11; MBL). In addition, a Tyramide Signal Amplification System (PerkinElmer, Waltham, Massachusetts) was used for SCF and IGF-1 detection in 16.5 dpc fetal spleen using appropriate dilutions of the following primary antibodies: anti-mouse SCF goat IgG (sc-1303; Santa Cruz Biotechnology) and anti-mouse IGF-1 goat IgG (AF791; R&D systems), both at 4°C overnight. After three PBS washes, sections were incubated with appropriate dilutions of the following secondary antibodies: Alexa Fluor®488 donkey anti-rat IgG (Invitrogen, Carlsbad, CA), HRP donkey anti-goat IgG (R&D systems), Alexa Fluor®546 Streptavidin (Invitrogen) as well as TOTO-3 (Invitrogen) to stain nuclei, at room temperature for 30 minutes. Samples were mounted on slides using fluorescent mounting medium (Dako Corporation), covered with cover glass, and assessed using a FluoView 1000 Confocal Microscope (Olympus).

### Co-culture of stromal and hematopoietic cells

For co-culture, whole fetal spleen cells were dissociated into single cells and cultured on 0.1% gelatin-coated tissue culture plate. After two hours, cells that had adhered to the plate were used as stromal cells. Non-adherent cells containing hematopoietic cells were collected and cultured with stromal cells in StemPro®-34 serum-free medium (SFM) (Gibco, Invitrogen, Carlsbad, CA) at 37°C and 5% CO_2_. After 24 hours of culture, cells were collected and assessed by flow cytometry after staining with the following antibodies: PB-conjugated anti-mouse CD45, APC-conjugated anti-mouse c-Kit, PE-conjugated anti-mouse CD71 and PE-Cy7-conjugated anti-mouse Ter119.

### *In vitro* functional assay

Whole fetal spleen cells were prepared as described above and cultured with StemPro®-34 SFM in the presence of the SCF inhibitor ISCK03 (10 µM) (sc-355981, Santa Cruz Biotechnology), the IGF-1R inhibitor PPP (10 µM) (sc-204008, Santa Cruz Biotechnology) or both (10 µM each). DMSO (Wako Pure Chemical Industries) served as a vehicle control at a final concentration of less than 0.1%. After 24 hours of culture, cells were stained with PB-conjugated anti-mouse CD45, APC-conjugated anti-mouse c-Kit and PE-Cy7-conjugated anti-mouse Ter119 and analyzed by flow cytometry.

### *In vivo* functional assay

Pregnant ICR mice at 16.5 dpc were intravenously injected with DMSO (control), the SCF inhibitor (1.56 µg/g body weight), the IGF-1R inhibitor (1.92 µg/g body weight) or both. Two hours later, fetal spleens from embryos were dissected and single cell suspensions were prepared as described.

### Statistical analysis

Data were presented as means±standard deviation (SD). Student's *t*-test was used to calculate statistical significance. A *P* value less than 0.05 was considered statistically significant.

## Supplementary Material

Supplementary Material
