# Supplementary material for: Localized SCF and IGF-1 secretion enhances erythropoiesis in the spleen of murine embryos
Source: Biol Open. 2015 Apr 17;4(5):596–607. doi: 10.1242/bio.201410686 (PMC4434811; doi:10.1242/bio.201410686)
Supplement: Supplementary Material [file supp_4_5_596__index.html]

Localized SCF and IGF-1 secretion enhances erythropoiesis in the spleen of murine embryos — Localized SCF and IGF-1 secretion enhances erythropoiesis in the spleen of murine embryos — Supplementary Material 

# Localized SCF and IGF-1 secretion enhances erythropoiesis in the spleen of murine embryos

## bio.201410686 Supplementary Material

**Files in this Data Supplement:**

- Supplementary Material - Keai Sinn Tan et al. doi: 10.1242/bio.201410686
