# Supplementary material for: Localized SCF and IGF-1 secretion enhances erythropoiesis in the spleen of murine embryos
Source: Biol Open. 2015 Apr 17;4(5):596–607. doi: 10.1242/bio.201410686 (PMC4434811; doi:10.1242/bio.201410686)
Supplement: Supplementary Material [file supp_bio.201410686_bio.201410686-s1.pdf]

Biology Open (2015) 000, 1–12 doi:10.1242/bio.201410686

Supplementary Material

Keai Sinn Tan et al. doi: 10.1242/bio.201410686

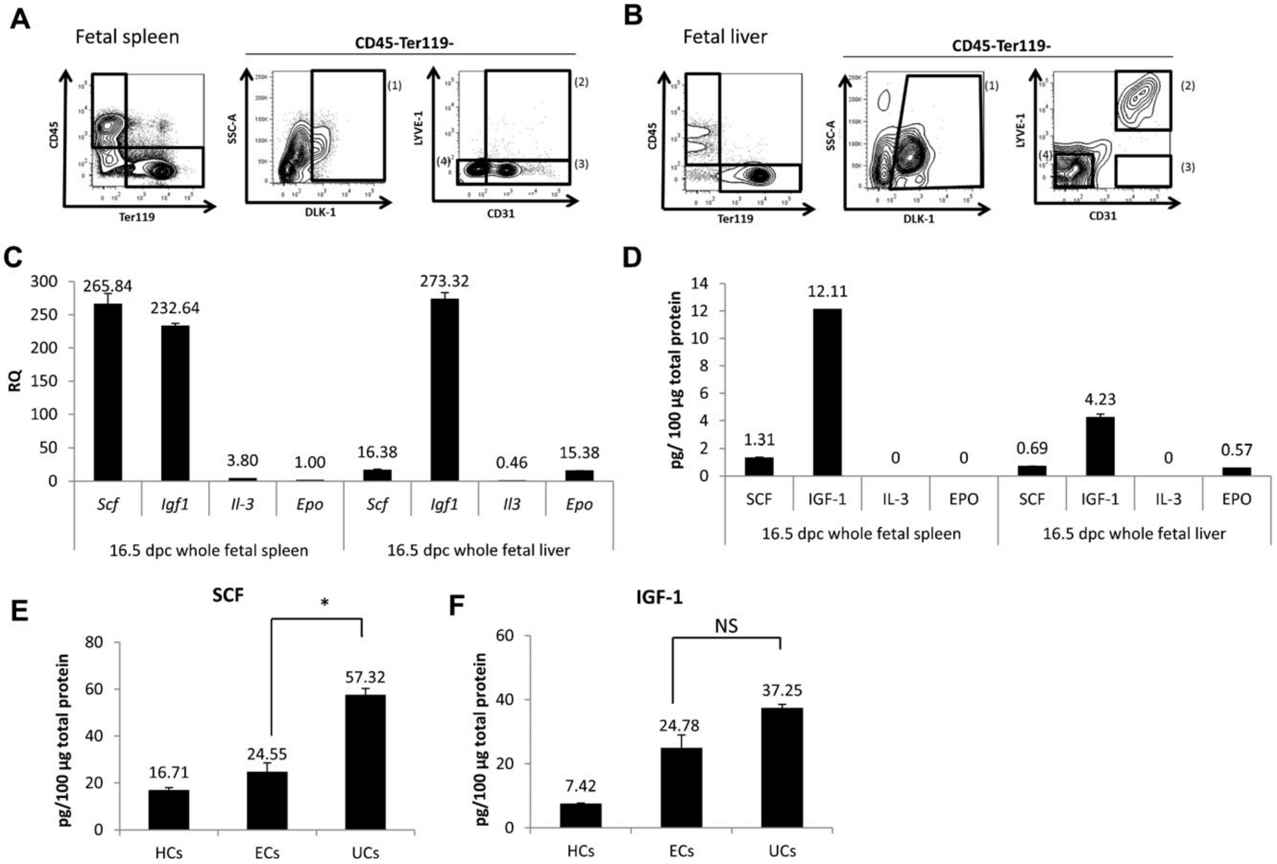

Fig. S1. Expression of SCF and IGF-1 in fetal spleen and liver. (A) Representative contour plots of flow cytometry of fetal spleen hematopoietic cells simultaneously labelled for the erythroid cell marker Ter119 and the common leukocyte cell marker CD45. CD45+Ter119−/CD45+Ter119+/CD45−Ter119+ defines hematopoietic cells (HCs). Spleen non-hematopoietic cells at 16.5 and 19.5 dpc were examined for expression of DLK-1, LYVE-1 and CD31 by flow cytometry; (1) CD45−Ter119−DLK-1+ defines DLK-1-expressing cells; (2) CD45−Ter119−CD31+LYVE-1+, microvessels; (3) CD45−Ter119−CD31+LYVE-1−, endothelial cells (ECs); and (4) CD45−Ter119−CD31−LYVE-1−, unclassified cells (UCs). (B) Contour plots of flow cytometry of fetal liver HCs labelled for both Ter119 and CD45. Liver non-hematopoietic cells at 16.5 and 19.5 dpc were examined for expression of DLK-1, LYVE-1 and CD31 by flow cytometry; (1) CD45−Ter119−DLK-1+ defines hepatoblasts; (2) CD45−Ter119−CD31+LYVE-1+, sinusoidal endothelial cells (SECs); (3) CD45−Ter119−CD31+LYVE-1−, ECs; and (4) CD45−Ter119−CD31−LYVE-1−, UCs. (C) Relative expression (RQ) of *stem cell factor* (*Scf*), *insulin-like growth factor1* (*Igf1*), *interleukin-3* (*Il-3*) and *erythropoietin* (*Epo*) mRNAs was assessed in whole fetal spleen and liver at 16.5 dpc by real-time PCR. Note high *Scf* and *Igf1* expression in whole fetal spleen and increased expression of *Igf1* relative to *Scf* in whole fetal liver. (D) Amounts of SCF, IGF-1, IL-3 and EPO protein per 100 μg total protein in both spleen and liver at 16.5 dpc. Note IGF-1 protein levels in whole fetal spleen exceed those in liver at 16.5 dpc. (n=3). (E,F) Amounts of SCF and IGF-1 protein per 100 μg total protein in HCs, ECs and UCs (n=3). Data are means±standard deviation (SD). NS, not significant. *P<0.05.

Biology Open (2015) 000, 1–12 doi:10.1242/bio.201410686

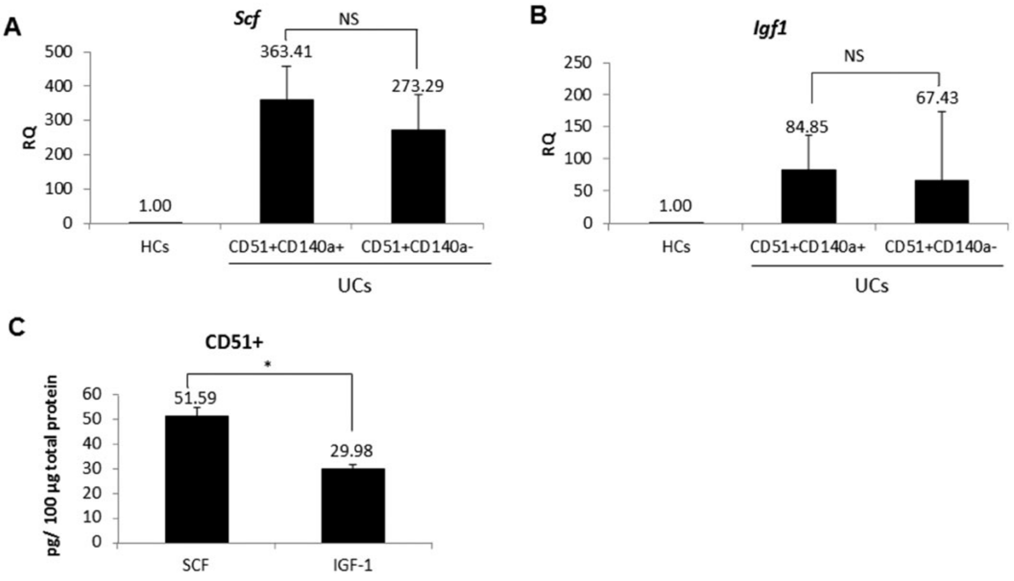

Fig. S2. Cytokine expression in CD51+ and CD51+CD140a+/− cells among unclassified cells (UCs). (A, B) Relative *Scf* and *Igf1* expression (RQ) was assessed by real-time PCR in hematopoietic cells (HCs) and CD51+CD140a+/− cells among UCs. HCs served as controls. *Scf* and *Igf1* expression was comparable in CD51+CD140a+/− cells compared to HCs. (n=3). (C) Amounts of SCF and IGF-1 protein per 100 µg of total protein in CD51+ cells. (n=3). Data are means±standard deviation (SD). NS, not significant. *P<0.05.

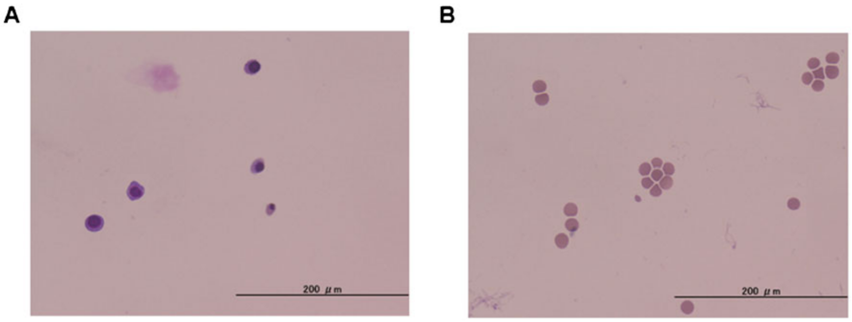

Fig. S3. Representative images showing the fetal spleen erythroid cell population. (A) May-Grünwald Giemsa staining of CD45−c-Kit−CD71+Ter119+ cells photographed at 400×. (B) Similar May-Grünwald Giemsa staining of the CD45−c-Kit−CD71−Ter119+ population photographed at 400×. Scale bars: 200 µm.

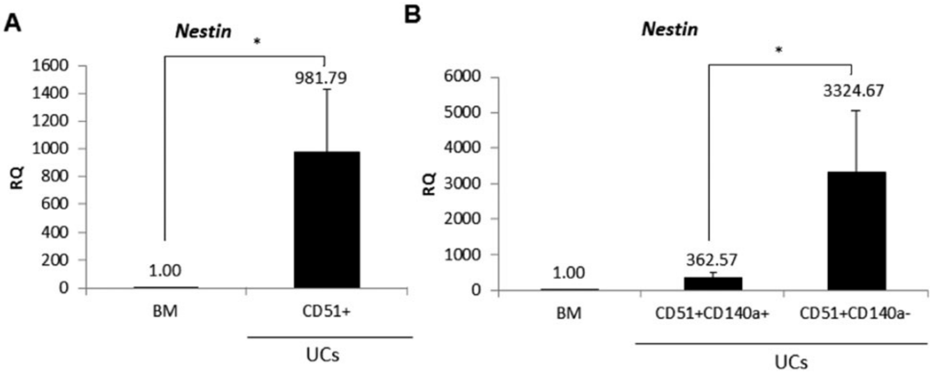

Fig. S4. *Nestin* expression in CD51+ and CD51+CD140a+/− cells among unclassified cells (UCs). (A) Relative *Nestin* expression (RQ) was examined by real-time PCR in control whole BM cells and in CD51+ cells. *Nestin* was expressed abundantly in CD51+ cells. (n=3). (B) *Nestin* expression was examined by real-time PCR in whole BM cells, CD51+CD140a+ and CD51+CD140a− cells. (n=3). *Nestin* expression was highest in CD51+CD140a− cells (n=3). Data are means±standard deviation (SD). *P<0.05.

Biology Open
